# Ultrastructure of compacted DNA in cyanobacteria by high-voltage cryo-electron tomography

**DOI:** 10.1038/srep34934

**Published:** 2016-10-12

**Authors:** Kazuyoshi Murata, Sayuri Hagiwara, Yoshitaka Kimori, Yasuko Kaneko

**Affiliations:** 1 National Institute for Physiological Sciences, Okazaki, Aichi, 444-8787, Japan; 2 Graduate School of Science and Engineering, Saitama University, Saitama, 338-8570, Japan; 3 National Institutes of Natural Sciences, Okazaki, Aichi, 444-8787, Japan

## Abstract

Some cyanobacteria exhibit compaction of DNA in synchrony with their circadian rhythms accompanying cell division. Since the structure is transient, it has not yet been described in detail. Here, we successfully visualize the ultrastructure of compacted DNA in the cyanobacterium *Synechococcus elongatus* PCC 7942 under rigorous synchronized cultivation by means of high-voltage cryo-electron tomography. In 3D reconstructions of rapidly frozen cells, the compacted DNA appears as an undulating rod resembling a eukaryotic condensed chromosome. The compacted DNA also includes many small and paired polyphosphate bodies (PPBs), some of which seem to maintain contact with DNA that appears to twist away from them, indicating that they may act as interactive suppliers and regulators of phosphate for DNA synthesis. These observations throw light on the duplication and segregation mechanisms of cyanobacterial DNA and point to an important role for PPBs.

Cyanobacteria are unicellular photosynthetic prokaryotes that produce about 25% of all carbohydrates on Earth; they have supplied the Earth with oxygen for more than two billion years^1^,^2^. They are also believed to be the ancestors of the chloroplasts found in plant cells^3^. Like other living cells, cyanobacteria face the problem of handling an enormous length of DNA within the limited space of the cell, necessitating a compact and functional packing method that permits the DNA to be transcribed, replicated, and divided at the appropriate times. Each 2–5 μm long rod-shaped cell of cyanobacterium *Synechococcus elongatus* PCC 7942 contains two to eight copies of circular chromosomal DNA consisting of approximately 2.7 Mbp, which means that each copy has a total length close to 1 mm^4^,^5^. Eukaryotic cells have developed sophisticated DNA packing mechanisms: the normal eukaryotic cell contains a nucleus and in cell division condensed chromosomes appear. Since Archaea form eukaryotic condensed chromosomes in cell division, they may be viewed as closer to eukaryotes^6^,^7^. Prokaryotic cells also show condensed DNA blobs or bundles, called nucleoid, in the cytoplasm, where ribosomes and multiple other complexes are effectively excluded to the periphery of the nucleoid^8^,^9^,^10^,^11^. However, this is not known to develop into a dramatic display of transformation on the occasion of cell division as in eukaryotic chromosomes^12^,^13^. For cyanobacteria it has been shown, using fluorescence microscopy, that DNA greatly changes appearance in *Synechococcus elongatus* PCC 7942 in synchrony with the circadian rhythm^14^. The fluorescent staining showed that DNA was usually uniformly located in the cytoplasm and was enclosed by thick layers of thylakoid membranes; however, when *S. elongatus* PCC 7942 was cultured under 12 hours light/12 hours darkness, its DNA appeared to become compacted at the end of the light period, whereas it was diffused throughout the cytoplasm at other times. Seki *et al*. have observed that in *S. elongatus* PCC 7942 actively growing on agar media, the DNA stained with Hoechst 33342 appears to be compacted into a distinct shape towards the end of the light period, when cell division occurs^15^. The DNA condensed gradually and showed a wavy rod-like shape under the light microscope just before cell division. This phenomenon may be related to bacterial DNA synthesis during the light period and with its subsequent distribution to daughter cells at the end of the light period^16^. However, it is very transient, unlike the persisting bacterial nucleoid, and thus the detailed structure of the compacted DNA has not been clarified yet.

*S. elongatus* PCC 7942 has the shape of a rod with a width of more than 0.5 μm in which compacted DNA appears for a very short time. To investigate the three-dimensional (3D) ultrastructure of the compacted DNA in the cell, it is necessary for electron microscopy to overcome two technical issues. One is the observation of a thick specimen and the other is the rapid fixation of a dynamical structure. First, the specimen penetration power of electrons depends on the accelerating voltage^17^. In the case of a regular transmission microscope (TEM) with under 300 kV accelerating voltage, penetration is generally limited to less than several hundred nanometers in biological specimens. To observe whole bacterial cells, a TEM using a higher accelerating voltage of over 500 kV is effective^18^. In this experiment, we adopted a high voltage electron microscope (HVEM) using 1 MV to observe the whole cells including the bacterial DNA. Second, cryo-electron tomography (cryoET) is a powerful tool to visualize the dynamical structure of biological specimens in 3D^19^, where one instant of a living cell is fixed by rapid freezing and directly observed by EM using a cryo-specimen holder. The 3D ultrastructure is computationally reconstructed from a tilt series after that.

For this study, we reproducibly induced DNA compaction in cyanobacteria by cultivation under a rigorous light/dark cycle, and identified the *in vivo* ultrastructure for the first time by means of high-voltage cryo-electron tomography assisted by rapid specimen freezing. The bacterial DNA formed a large and undulating rod-shaped body, resembling a eukaryotic condensed chromosome, toward the end of the light period. This rod-shaped body contained many small and paired polyphosphate bodies (PPBs), some of them attached to DNA that appears to twist away, indicating that they probably act as interactive suppliers and regulators of phosphate for DNA synthesis.

## Results

### Fluorescence Microscopy

**Figure 1:**
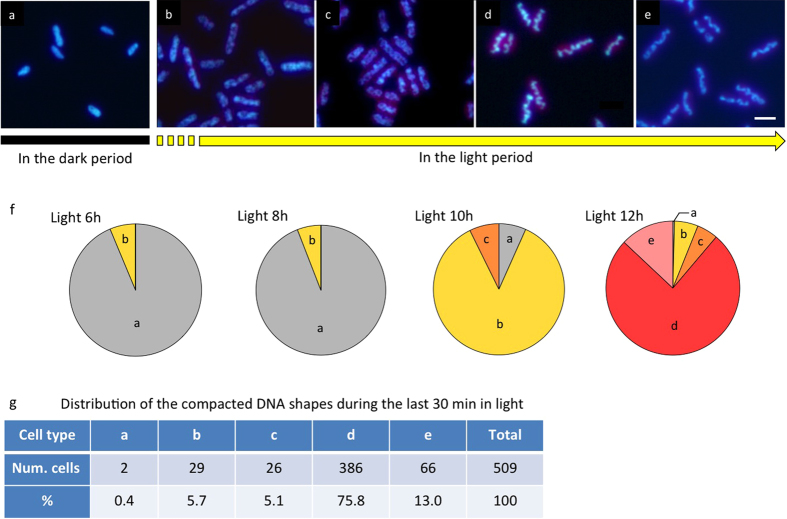
Fluorescence microscopy of cyanobacterium *S. elongatus* PCC 7942 cells. Cells were cultured under 12 h light/12 h dark cycles and stained with Hoechst 33342. Red color indicates auto fluorescence of thylakoid membranes in the cells. (**a**) Cells 2 h after the onset of the dark condition, showing uniform DNA labeling. The overall DNA structure of a population of cells gradually changes during the light period to give numerous clumps of condensed DNA (**b**); these, in turn, change into spiral or ring-like strings (**c**). Subsequently, they form a thick wavy rope-like structure (**d**), representing typical DNA compaction. The compacted DNA structures finally separate at their centers (**e**) and the cells divide. Scale bar: 2 μm. (**f**) The fractions of DNA structures in forms (**a**) to (**e**) from 6 to 12 h of the light period. (**g**) The distribution of DNA in forms (**a**) to (**e**) during the final 30 min under light conditions.

Vigorously growing *S. elongatus* PCC 7942 cells cultured under a 12 hours light/12 hours dark regime were observed by fluorescence microscopy after labeling with Hoechst 33342 (Fig. 1). Cells cultured for two hours in the dark period of the cycle showed uniform DNA labeling (Fig. 1a). In contrast, cells that had been cultured for 12 hours in the light (just before onset of the dark period), contained a wavy rod-like structure of bacterial DNA, indicating DNA compaction (Fig. 1d). The compacted DNA then separated at the middle (Fig. 1e) and was distributed into the daughter cells. This phenomenon was transitory, but we could observe several intermediate steps in the development of the compacted DNA as a result of cultivation under the rigorous light/dark cycle. At first, the DNA, which was previously diffused throughout the cells, became partly condensed and formed several clumps (Fig. 1b); it then changed into strings that adopted spiral or ring shapes (Fig. 1c). This sequence of changes, as shown in Fig. 1a–e, occurred during the light period, with time delays between various cell populations on one plate (Fig. 1f). In particular, at the end of the light period, the stained DNA exhibited a dramatic change so that more than 90% of the cells were in a state corresponding to one of those shown in Fig. 1b–e,g. The special structure disappeared immediately after cell division, at the end of the light period, and the DNA returned to a uniform distribution (Fig. 1a). Under this exact culture condition, a similar phenomenon also occurred in the dark period, but the fraction of cells showing these features was low (Supplementary Fig. 1).

### High voltage cryo-electron tomography

**Figure 2:**
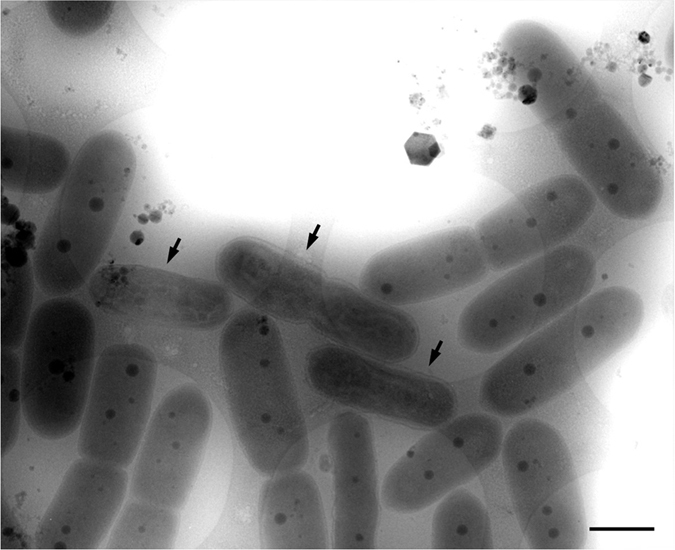
High-voltage electron microscope image of the ice-embedded cyanobacteria. Images were taken at 12 h under light conditions. Some cells show undulating eukaryotic condensed chromosome-like rod-shaped DNA bodies (arrows). In normal cells, cyanobacteria show a uniform contrast of DNA in the cytoplasm. The cytoplasm contains one or two large distinct polyphosphate bodies (PPBs) in addition to several small ones (high density dots). Scale bar: 1 μm.

**Figure 3:**
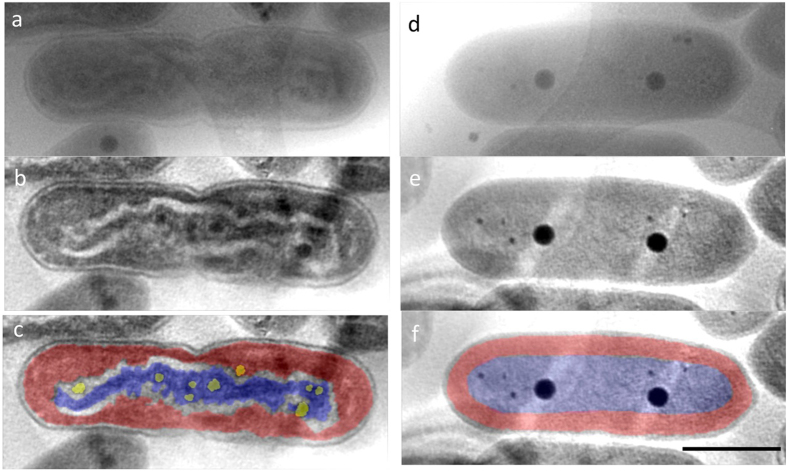
High-voltage electron tomograms of ice-embedded cyanobacteria. The tilt series for tomography was collected from the same area as in Fig. 2, and subjected to a 3D reconstruction. (**a**) Untilted images of a cell that contains compacted DNA. (**b,c**) A *z*-axis slice of the tomographic reconstruction in the area shown in (**a**), showing wavy undulating rod-shaped DNA bodies (colored blue in **c**). The thylakoid membrane layers and PPBs are colored red and yellow, respectively (**c**). (**d–f**) the counterpart views of (**a–c**) in a normal cell. In normal cells, cyanobacteria show a uniform contrast of DNA (colored blue in **f**) in the cytoplasm surrounded by thylakoid membrane layers (colored by red in **f**). The cytoplasm contained one or two large distinct polyphosphate bodies (PPBs) in addition to several small ones (black blobs in **d–f**). Compared to normal cells, in the cells containing compacted DNA, the central compacted DNA is clearly separated from the peripheral thylakoid membranes by low-density cytoplasm and the PPBs are smaller than those in normal cells and some of them occur as pairs. Scale bar: 1 μm.

By means of high-voltage electron microscopy (HVEM) with 1 MV accelerating voltage to ensure that the electrons penetrated the specimens, which in some cases were more than 1 μm thick^17^,^18^, we were able to observe the fine structure of the compacted DNA in cyanobacteria. Specimens that most clearly showed DNA compaction under the light microscope at 12 hours of the light period of the cycle were rapidly frozen and embedded in thin amorphous ice on holey-carbon coated EM grids. Cryo-electron tomography using the HVEM permitted visualization of the 3D ultrastructure of the entire cyanobacterial cells, including the compacted DNA, thylakoid membrane layers, cell walls, and polyphosphate bodies (PPBs), in their near-native state^20^ (Fig. 2). Supplementary Movies 1 and 2 show HVEM tilt-series images of ice-embedded cyanobacteria and *z*-axis slices of the tomographic reconstruction, respectively. Some of these images clearly show the DNA compaction to form a nucleoid of undulating shape (arrows in Figs 2 and 3a–c). Areas of low-density cytoplasm that separate the chromosomal DNA (blue label in Fig. 3c) from the thylakoid membranes (red label in Fig. 3c) suggest that the compacted DNA formed a highly-condensed eukaryotic chromosome-like structure in the cytoplasm. In the compacted DNA, a large number of small and weak-density PPBs were observed (yellow blobs in Fig. 3c). In normal cells, on the other hand, cyanobacteria showed a low and uniform contrast of DNA in the cytoplasm (blue label in Fig. 3f), which tended to include one to two prominent PPBs in addition to several smaller ones (dark blobs in Fig. 3f). It was difficult to distinguish the dispersed nucleoid from the cytoplasm. However, thylakoid membranes were observed as a uniform multilayer between the cell wall and the cytoplasm (red label in Fig. 3f), although each layer was not very distinct because only low contrast could be achieved with the unstained specimens (Fig. 3d,e).

### Ultrastructure of major organelles

**Figure 4:**
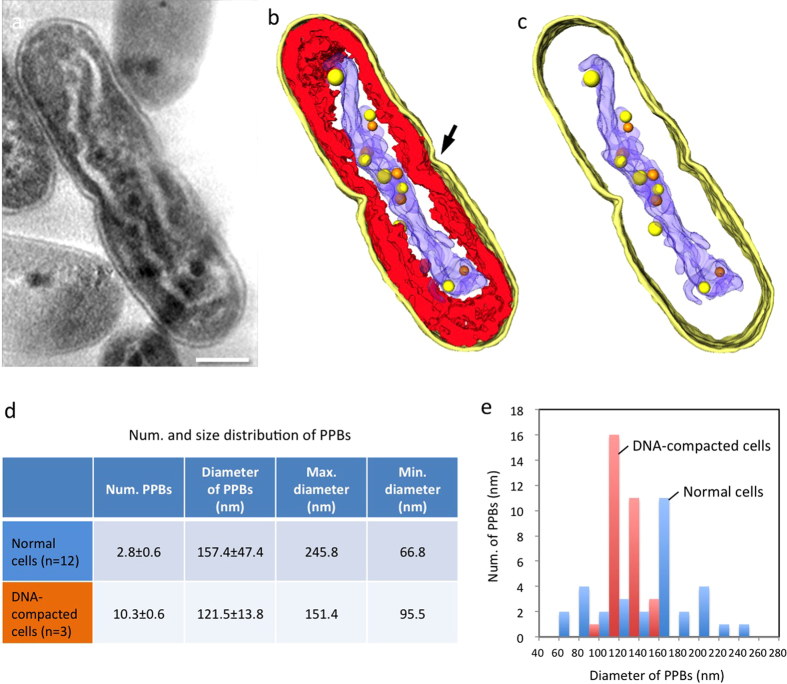
The ultrastructure of cells containing compacted DNA. The major components are segmented in the reconstructed tomogram: cell wall (bright yellow), thylakoid membranes (red), and DNA (blue). (**a**) shows a z-slice of the 3D tomogram. (**b**) all segments. In (**c**) the thylakoid membrane is omitted, and only the cell wall and the PPBs are presented. In the initial stages of cell division, a structure indicative of cell separation appeared in the middle of the longer axis of the cell (arrow in **b**). PPBs are modeled as yellow or orange spheres; each orange sphere represents an apparent companion of the closest yellow one. (**d**) Table of the numbers and size distributions of PPBs in normal cells and in those containing compacted DNA. (**e**) Size distribution of PPBs in both types of cell. PPBs were smaller and had constant sizes in compacted-DNA cells (red), but they occurred in markedly higher numbers than in normal cells (blue).

We segmented the structures of major organelles in the cells containing compacted DNA from the reconstructed tomogram and displayed them separately (Fig. 4 and Supplementary Movie 3). In normal cells, the thylakoid membrane layers were uniformly distributed between the cell wall and the low-density cytoplasm, as observed by means of the conventional defocus contrast technique^21^ (Fig. 3d–f and Supplementary Movies 1 and 2). By contrast, in cells containing compacted DNA, thylakoid membrane layers were significantly distorted and surrounded the central compacted DNA across a region of low-density material (Figs 3a–c and 4a,b). There were some areas in which the undulating compacted DNA was closely associated with neighboring thylakoid membrane layers. In the case of cells in the initial stage of cell division, a structure indicating incipient separation appeared in the middle of the longer axis of the cell (arrow in Fig. 4b). The compacted DNA extended continuously to both sides of the future daughter cells, and was not yet separated.

### Polyphosphate bodies

**Figure 5:**
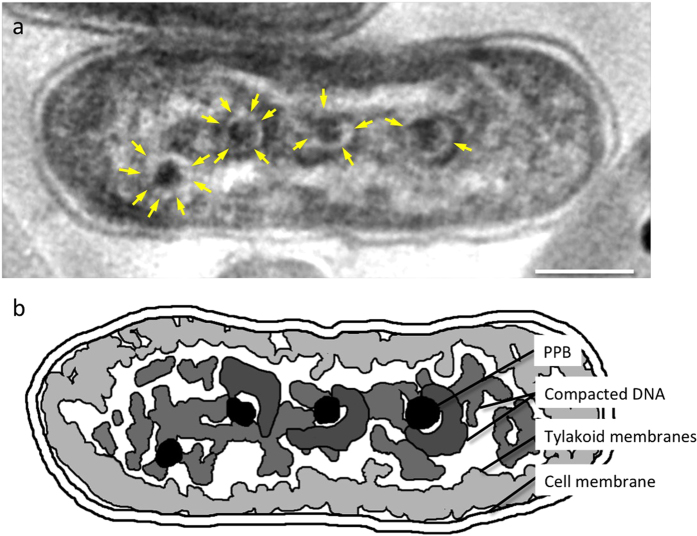
A magnified image of the compacted DNA and PPBs in the cyanobacterial cell. (**a**) A z-slice of the 3D tomogram. The compacted DNA holds PPBs, and the edge of the DNA connects to the PPBs through several thin threads (yellow arrows). Some of the DNA looks as if it is twisting away from a PPB (darker gray). (**b**) Schematic representation of (**a**). Scale bar: 500 nm.

Under these sampling conditions, the normal cells observed were assumed to be newly born cells after cell division; here 1–3 conspicuous PPBs, which were ~160 nm in diameter, were observed in addition to several smaller ones with a diameter of ~80 nm (black dots in Fig. 3d–f, blue bars in Fig. 4e). In contrast, the structures observed showed that cells exhibiting DNA compaction contained relatively small and weak-density PPBs (yellow labels in Fig. 3c), which were not observed in a previous study^15^. As a result of the statistical analysis, all PPBs in the DNA compacted cells were of similar sizes (121.5 ± 13.8 nm) and were smaller than those in normal cells (157.4 ± 47.4 nm); furthermore, they appeared in markedly greater numbers (10.3 ± 0.6) than the number of large PPBs in normal cells (2.8 ± 0.6) (Fig. 4d,e). In the cells with compacted DNA, almost all the PPBs were contained in and attached to the compacted DNA and they were often observed as pairs. The orange spheres shown in Fig. 4b,c are the counterparts of the nearby yellow spheres in PPB pairs. Vestiges of pairs of PPBs were also observed in the plastic ultra-thin section, although the corresponding PPBs had been lost during sample preparation (Supplementary Fig. 2a). These observations show that pairs of PPBs appear in the cells containing compacted DNA before cell division, suggesting that the PPBs may divide during DNA compaction, presumably corresponding to DNA rearrangement and segregation. In addition, in the cells containing compacted DNA, the chromosomal DNA was closely associated with the edges of PPBs (Fig. 5 and Supplementary Fig. 2b), where some of the DNA seemed to be twisting away from a PPB (darker gray label in Fig. 5b). Actually, the compacted DNA holding PPBs was connected to the PPBs through several thin threads or strands (arrows in Fig. 5a and Supplementary Fig. 2c,d,f). This microscopic observation of PPBs suggests that they may function as phosphate suppliers during DNA synthesis.

## Discussion

In this study, to identify the ultrastructure of compacted DNA in specially prepared cyanobacterial cells, we used rigorous cultivation under light/dark cycles and high voltage cryo-electron tomography after rapid specimen freezing. We successfully captured and visualized the transient structure of the compacted DNA in the cells. This revealed several new features in cyanobacteria during cell duplication.

The structure of the DNA compaction in cyanobacteria can be distinguished from the nucleoids in bacteria in some points. The nucleoids in bacteria show as parallel blobs or bundles of DNA, sometimes twisting, and they are not extremely transient^8^,^9^,^10^,^11^. By contrast, the DNA compaction of cyanobacteria is very transient and the structure drastically changes as cell division occurs. In the normal cell, the DNA is uniformly distributed in the cytoplasm, which is closely facing the thylakoid membrane layers as shown in Fig. 3d–f. When the DNA compaction happens, the condensed DNA excludes the low-density material. Eventually, the compacted DNA is separated from the thylakoid membranes by a layer of low-density material (Figs 3a–c, 4a,b and 5). The ribosomes and numerous other complexes are presumably absorbed by the compacted DNA or the peripheral thylakoid membranes. This suggests that the compacted DNA in cyanobacteria develops according to mechanisms that differ in several respects from the features generally found in bacterial nucleoids.

For bacteria, the duplicated DNA is anchored to the cell membrane and separated into the two daughter cells by the movement associated with cell elongation^22^. In cyanobacteria, thylakoid membrane layers appear between the plasma membrane and cytoplasm^23^. Ting *et al*. have investigated the structure of the small cyanobacterium *Prochlorococcus* by electron cryo-tomography at 300 kV and revealed the uniform layer structure of its thylakoid membranes^24^. In *Synechococcus* cells, the existence of a uniform layer structure has been identified by Hilbert-phase contrast electron microscopy (HDC-EM) with ice-embedded cells and by regular EM with ultra-thin plastic sections stained with heavy metals^25^,^26^. It is believed that the DNA is anchored to the thylakoid membrane layer and segregated according to the elongation in cyanobacteria. However, the cyanobacterial cells including the compacted DNA present a clear separation between the DNA and the thylakoid membranes in our observation. The distorted layers of the thylakoid membranes partly connected with the compacted DNA may preserve the function of the bacterial cell membrane in migrating the divided DNA into individual daughter cells.

The cytoplasm of most cyanobacteria contains prominent PPBs^27^, but their roles remain poorly understood because it is difficult to observe their fine structure by regular EM using ultrathin sections. Since PPBs are not sufficiently infiltrated with resin compared with other organelles, larger PPBs are fragile and easily lost during ultrathin sectioning (Supplementary Fig. 2)^15^. HDC-EM has been used to observe PPBs and other structures directly in near-native cyanobacterial cells embedded in ice^28^,^29^. However, the procedure intrinsically involves technical artifacts in the image, and thus no further interpretation of the three-dimensional fine structure has been achieved. Here, we used high-voltage cryo-electron tomography to successfully observe the transient PPBs, and to determine their numbers, sizes, and locations in the cytoplasm, in addition to their dynamics.

In our previous work, changes in the numbers and sizes of PPBs during DNA compaction were observed by light microscopy^15^. The size of PPBs stained with dye increased in diameter from roughly 300 nm to 400 nm during the dark period, and reverted to 300 nm during the light period. The number of stained PPB spots doubled from 1~2 to 2~4 in the light period of the normal cells. Stained PPBs could not, however, be detected in the DNA-compacted cells. Here, we accurately measured the size and the number of PPBs in both normal and DNA-compacted cyanobacteria at the end of the light period. As a result, we newly identified small and paired PPBs in the DNA compacted cells. The number was approximately four times higher than in the normal cells. The presence of ~10 PPBs in the cells containing compacted DNA is reasonable, if we assume a two-step division model for PPBs in which, for example, 1~2 PPBs increase to 2~4 PPBs in normal cells during the light period and these further form pairs to finally give a total of 4~8 PPBs in the compacted DNA at the end of the light period. In our observation, we detected ~3 PPBs in the normal cells and 10 or more PPBs in the cells containing compacted DNA. The small satellite PPBs observed in the normal cells may also contribute to the fluctuations in the numbers of PPBs. It is known that the copy number of the DNA ranges from 2 to 8 in cyanobacteria and is correlated with the cell length^5^,^30^. The copy number of the DNA may, therefore, be correlated to the number of PPBs.

Seki *et al*. have reported that the volume of individual PPBs reduces during the light period in the synchronous culture, where DNA duplication and DNA compaction progress toward the end of the light period when finally cell division occurs^15^. They also observed that PPBs are connected to fibers containing cyanobacterial DNA, which was confirmed in ultra-thin sections stained with osmium–ammine, which specifically labels nucleotides. However, it was not entirely clear that the appearance of the fibers is not, at least partially, an artifact caused by PPB shrinkage in the chemically fixed cells. Here, we observe some twisting densities and several protrusions extending from the PPBs in the rapidly frozen cells containing compacted DNA (Fig. 5). In addition to the mysterious behavior of PPBs during the light period, this significantly strengthens the claim that phosphate for the synthesis of DNA is interactively supplied and regulated by PPBs, resulting in the division and reduction in volume of the PPBs, although more study is needed to prove this hypothesis.

In summary, we have positively induced DNA compaction in *S. elongatus* PCC 7942 by vigorous culture under 12 hours light/12 hours dark cycles. We have detected and observed the DNA compaction in cells by means of high voltage cryo-electron tomography with the aid of rapid specimen freezing. This allowed us to determine the 3D ultrastructure of the cells containing compacted DNA and provides strong evidence of interactive association between the compacted DNA and PPBs. The DNA compaction observed in cyanobacteria reminded us of the formation of condensed chromosomes in eukaryotes, which provides safe packing and equal division of the genome into daughter cells. Our observations raise the question of whether the cyanobacteria may have the potential to form highly condensed chromosomes like those of eukaryotes. It has been reported that cyanobacteria have several DNA-binding proteins that have no homology with those in eukaryotes^31^, and that the mechanisms of DNA folding and DNA segregation are probably quite different from those in eukaryotes. However, our observations of the DNA compaction in cyanobacteria suggest that the compacted DNA could be a primal version of the formation of the eukaryotic condensed chromosome. Therefore, exploration of the exact mechanism of cyanobacterial DNA compaction may provide new insight into the evolution of DNA folding and the formation of condensed chromosomes. Our work also suggests that PPBs are interactively involved in DNA duplication and compaction. Further studies of DNA compaction and PPB formation should shed light on the evolution of condensed chromosome formation in eukaryotes.

## Methods

### Cyanobacterial strain and culture conditions

*Synechococcus elongatus* PCC 7942 was provided courtesy of Dr. Hitoshi Nakamoto, Saitama University. The cells were cultured at 23 °C under a light intensity of 50 μE/m^2^/s with 12 h light/12 h dark cycle on BG 11 plates containing 1.5% (w/v) agar and 0.3% (w/v) sodium thiosulfate^32^. The cells were transferred to fresh media every week, and the sixth-day culture was used for observation of DNA compaction.

### Sample preparation for electron microscopy

For fluorescence labeling of the DNA, the collected cells were stained with Hoechst 33342 at a final concentration of 1 μg/mL for 10 min in the dark. The cells were observed with a Nikon ECLIPSE 50i fluorescent microscope (Nikon, Tokyo, Japan) equipped with a V-2A filter. The cells that showed DNA compaction before cell division at the end of the light cycle were rapidly frozen and ice-embedded for electron cryo-microscopy, in addition to the conventional chemical fixation and resin embedding described previously^15^. To prepare the ice-embedded cyanobacteria, 2.5 μl aliquots of the collected cells were mounted on Quantifoil copper grids R3.5/1 (Quantifoil MicroTools, Jena, Germany) pretreated by glow-discharge. Excess solution was removed by blotting with a filter paper, and the samples were manually rapidly frozen in liquid ethane by a LEICA plunge-freezing device (LEICA EM CPC, Leica, Vienna, Austria). The frozen grids were stored in liquid nitrogen until required.

### High-voltage electron cryo-tomography

The grid with the ice-embedded cells was set on a cryo-specimen holder for HVEM (Gatan Inc. Pleasanton, CA, USA) precooled with liquid nitrogen to –150 °C. They were then transferred into a high-voltage H-1250M electron microscope (Hitachi Co. Ltd., Tokyo, Japan) operated at 1 MV accelerating voltage. The tilt series were collected from −60° to +60° in 3° steps. The images were recorded at a magnification of ×10,000 on electron films (Kodak SO-163) with a low electron dose to minimize specimen damage. Underfocus of 6 to 10 μm was used. The negatives were developed with full-strength D-19 developer (Kodak) for 12 min and subsequently digitized using a Coolscan 9000ED flatbed scanner (Nikon Co. Ltd., Tokyo, Japan) at a resolution of 4000 dpi. The image data were finally reduced in size by using a median filter with binning 2, representing a resolution of 1.27 nm/pixel.

### Image processing

Series of tilted images were processed with IMOD software^33^, and reconstructed into 3D tomograms. The 3D images were processed with a mathematical morphology filter to reduce noise and to extract cellular components^34^. The filtered images were used to manually segment the structures with Amira software (FEI Visualization Science Group, Burlington, MA, USA).

## Additional Information

**How to cite this article**: Murata, K. *et al*. Ultrastructure of compacted DNA in cyanobacteria by high-voltage cryo-electron tomography. *Sci. Rep.* **6**, 34934; doi: 10.1038/srep34934 (2016).

## Supplementary Material

Supplementary Information

Supplementary Movie S1

Supplementary Movie S2

Supplementary Movie S3
