# Supplementary material for: Ultrastructure of compacted DNA in cyanobacteria by high-voltage cryo-electron tomography
Source: Sci Rep. 2016 Oct 12;6:34934. doi: 10.1038/srep34934 (PMC5059737; doi:10.1038/srep34934)
Supplement: Supplementary Information [file srep34934-s1.pdf]

Supplementary information

Ultrastructure of compacted DNA in cyanobacteria by high-voltage cryo-electron tomography

Kazuyoshi Murata[1,*], Sayuri Hagiwara[2], Yoshitaka Kimori[3], Yasuko Kaneko[2,*]

[1]National Institute for Physiological Sciences, Okazaki, Aichi, 444-8787, Japan

[2]Graduate School of Science and Engineering, Saitama University, Saitama, 338-8570, Japan

[3]National Institutes of Natural Sciences, Okazaki, Aichi, 444-8787, Japan

*Corresponding authors: kazum@nips.ac.jp (KM); yakaneko@mail.saitama-u.ac.jp (YKa)

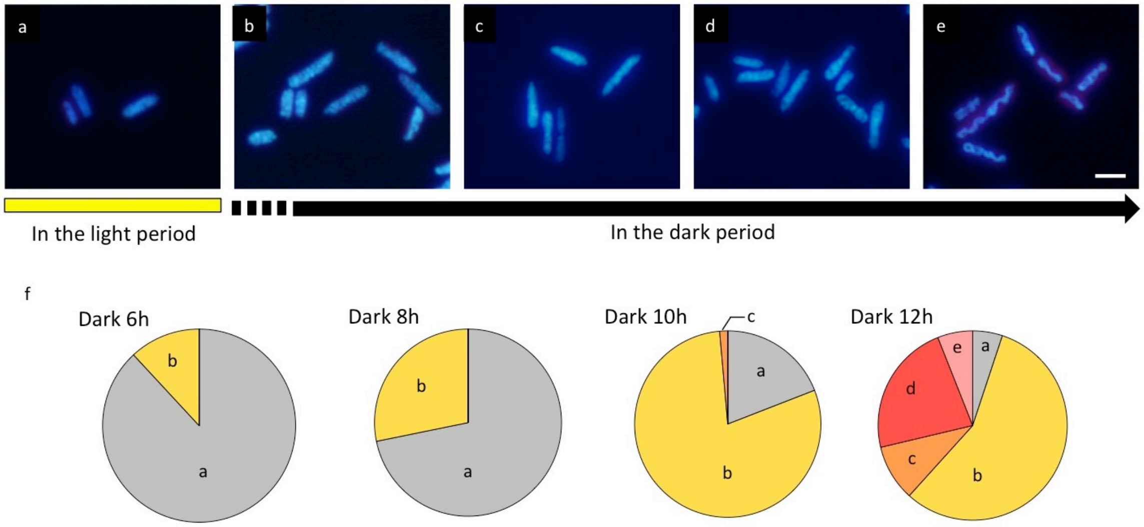

Supplementary Figure 1. Fluorescence microscopy of cyanobacterium *S. elongatus* PCC 7942 cells during the dark period. Cells were cultured under 12 h light/12 h dark cycles and stained with Hoechst 33342. (**a**) Cells 2 h after the onset of the light condition, showing uniform DNA labeling. The overall DNA structure of cells in the cell populations gradually changes during the dark period, approximating the wavy rope-like structure that appears during the light period. Scale bar: 2 µm. (**f**) The fractions of DNA structures in forms (**b**) to (**e**) from 6 to 12 h of the dark period.

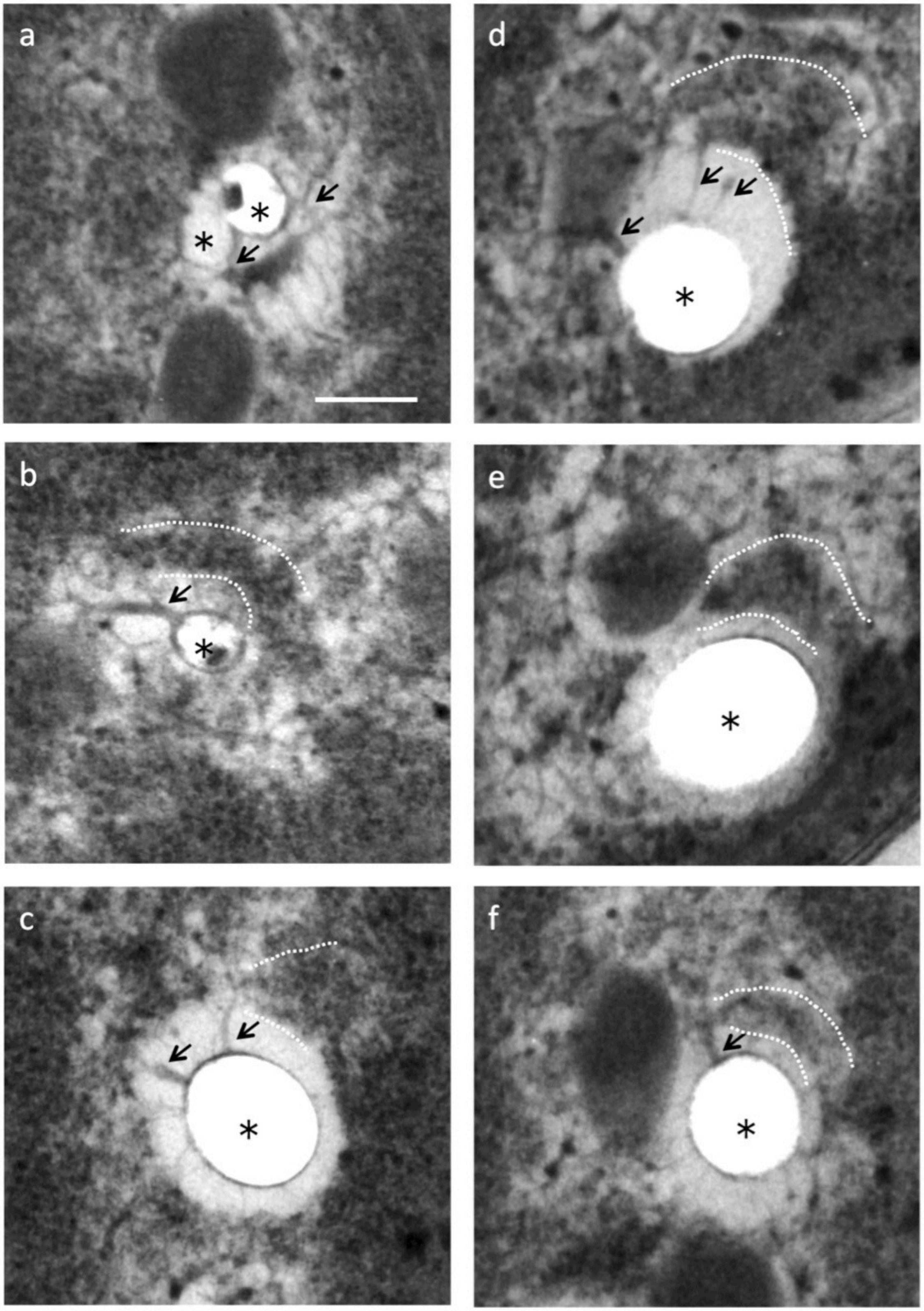
a
b
c
d
e
f

Supplementary Figure 2. Electron micrographs of the vestiges of PPBs in chemically fixed and resin-embedded sections of *S. elongatus* PCC 7942 cyanobacteria that showed DNA compaction. PPBs were lost during sample preparation, leaving holes in the sections (asterisks). (**a**) The holes left by a pair of PPBs or possibly by a PPB dividing in two. (**b-f**) The compacted DNA (between the dotted lines) held a PPB, and some thread-like connections between the compacted DNA and the PPB (arrows) remain visible. Scale bar: 100 nm. For conventional chemical fixation, the cells were collected from agar plates with 0.2 M sucrose solution and centrifuged (2000 g for 1 min) to make pellets. The pellets were fixed in 2% glutaraldehyde in 0.05 M potassium phosphate buffer (pH 7.0) for 2 h at room temperature and in a refrigerator overnight. After rinsing in the buffer, the cells were post fixed with 2% OsO_4 in the buffer for 2 h at room temperature. They were then dehydrated in an acetone series and embedded in Spurr's resin. Ultrathin sections (silver-gold color, ca. 90 nm in thickness) were cut with a diamond knife on a Sorvall MT2-B ultra-microtome. After staining with uranyl acetate and lead citrate, the sections were observed with a Hitachi H-7500 TEM at an accelerating voltage of 100 kV. The images were recorded on electron microscope films (Kodak 4489) at a magnification of ×10,000 with underfocus of 1 to 2 μm. The films were developed with full-strength D-19 and the images were digitized by a scanner (Canoscan 9950F).

Supplementary Video 1. Tilt-series images of cyanobacterium *S. elongatus* PCC 7942 for high-voltage electron tomography.

Supplementary Video 2. *Z*-slices of the tomographic 3D reconstruction of cyanobacterium *S. elongatus* PCC 7942.

Supplementary Video 3. Segmentations of major organelles in *S. elongatus* PCC 7942 cells.
